# Murine Model of Sinusitis Infection for Screening Antimicrobial and Immunomodulatory Therapies

**DOI:** 10.3389/fcimb.2021.621081

**Published:** 2021-03-12

**Authors:** Morgan A. Alford, Ka-Yee G. Choi, Michael J. Trimble, Hamid Masoudi, Pavneet Kalsi, Daniel Pletzer, Robert E. W. Hancock

**Affiliations:** ^1^ Centre for Microbial Diseases and Immunity Research, University of British Columbia, Vancouver, BC, Canada; ^2^ British Columbia Centre for Disease Control, Public Health Services Authority, Vancouver, BC, Canada; ^3^ Department of Pathology and Laboratory Medicine, University of British Columbia, Vancouver, BC, Canada; ^4^ Department of Microbiology and Immunology, University of Otago, Dunedin, New Zealand

**Keywords:** antimicrobial, host-defense peptides, inflammation, *Staphylococcus aureus*, *Pseudomonas aeruginosa*, sinusitis, therapies screening

## Abstract

The very common condition of sinusitis is characterized by persistent inflammation of the nasal cavity, which contributes to chronic rhinosinusitis and morbidity of cystic fibrosis patients. Colonization by opportunistic pathogens such as *Staphylococcus aureus* and *Pseudomonas aeruginosa* triggers inflammation that is exacerbated by defects in the innate immune response. Pathophysiological mechanisms underlying initial colonization of the sinuses are not well established. Despite their extensive use, current murine models of acute bacterial rhinosinusitis have not improved the understanding of early disease stages due to analytical limitations. In this study, a model is described that is technically simple, allows non-invasive tracking of bacterial infection, and screening of host-responses to infection and therapies. The model was modified to investigate longer-term infection and disease progression by using a less virulent, epidemic *P. aeruginosa* cystic fibrosis clinical isolate LESB65. Tracking of luminescent bacteria was possible after intranasal infections, which were sustained for up to 120 h post-infection, without compromising the overall welfare of the host. Production of reactive oxidative species was associated with neutrophil localization to the site of infection in this model. Further, host-defense peptides administered by Respimat^®^ inhaler or intranasal instillation reduced bacterial burden and impacted disease progression as well as cytokine responses associated with rhinosinusitis. Thus, future studies using this model will improve our understanding of rhinosinusitis etiology and early stage pathogenesis, and can be used to screen for the efficacy of emerging therapies pre-clinically.

## Introduction

*Pseudomonas aeruginosa* is an opportunistic pathogen that is a major cause of nosocomial and eventually fatal respiratory tract infections in cystic fibrosis (CF) patients (Bara et al., 2018). Individuals with CF are highly susceptible to respiratory infections due to copious production of mucus that reduces ciliary function and the effectiveness of innate immune responses (Smith et al., 2006; Winstanley et al., 2016). One-third of *P. aeruginosa* CF infections occur within the first 6 months of life and, despite reports of transmission between patients, the majority are caused by environmental isolates (Li et al., 2005; Jelsbak et al., 2007). Although many studies have examined changes in bacterial diversity associated with long-term adaptation to the lung environment during chronic infection, relatively little is known about the early stages of *P. aeruginosa* colonization and pathogenesis. Initial colonization of the nasopharynx and paranasal sinuses has been suggested to facilitate incremental adaptation and physiological optimization for the anoxic conditions characteristic of the CF lung (Aanaes et al., 2011; Fothergill et al., 2014). The genetic similarity of bacteria isolated from the upper and lower airways over time further suggests that the nasal cavity is a reservoir for recurrent *P. aeruginosa* and *Staphylococcus aureus* lung infections (Mainz et al., 2009). It has been reported that 60%–80% of CF patients have radiographic evidence of rhinosinusitis, but less than 20% of patients report relevant symptoms (Berkhout et al., 2014; Tipirneni and Woodworth, 2017).

Rhinosinusitis is a multifactorial disease characterized by inflammation of the sinus mucosa (Hoggard et al., 2017). Symptoms of this disease may include, but are not limited to, nasal congestion, discharge and obstruction, facial pain, edema or obstruction of sinuses (Fokkens et al., 2012; Orlandi et al., 2016). The etiology of rhinosinusitis can involve host and/or environmental factors, including sinonasal anatomic abnormalities, scarring of the sinus cavity due to prior injuries, or genetic disorders that cause immunodeficiency or autoimmune diseases, fungal or bacterial infection, allergy or pollutants (Fastenberg et al., 2016). Since this disease is not well defined, diagnosing patients is very challenging. Despite this, rhinosinusitis is one of the most common medical complaints in North America, affecting 12.5%–16% of the population (Meltzer and Hamilos, 2011; Schleimer, 2017). In the United States, rhinosinusitis accounts for approximately 24 million physician visits (Meltzer and Hamilos, 2011), with an estimated aggregated cost of more than $8 billion USD per year (Schleimer, 2017). Chronic rhinosinusitis, characterized by persistent symptoms, is particularly troubling since it is often associated with bacterial biofilm infections, which are recalcitrant to therapy and recur even after surgical debridement or antibiotic therapy. Chronic rhinosinusitis contributes to 4.1 million physician visits and 242,000 emergency room visits annually, and greatly impacts the quality of life in patients (CDC, 2017). Prince et al. (2008) obtained sinonasal cultures from 157 chronic rhinosinusitis patients over 4 months and found that 71% of samples comprised *S. aureus* and/or *P. aeruginosa*. A more recent study examined isolates from chronic rhinosinusitis patients who underwent endoscopic sinus surgery and showed that the most common species were Staphylococci (28%), followed by *Streptococcus* (12.2%), *Propionibacterium* (8%), *Haemophilus* (7.5%), *Klebsiella* (5.1%), and *P. aeruginosa* (4.2%) (Kim et al., 2019). Kim et al. also showed that the proportion of Staphylococci, *Klebsiella* and *P. aeruginosa* isolated from these patients significantly increased over time (Kim et al., 2019).

Multiple immune mechanisms exist at the mucosal surface to influence host-pathogen interactions and may contribute to the aberrant inflammation associated with this condition (Lee and Lane, 2011). Thus, a diverse range of factors have been investigated as drivers of rhinosinusitis, including defects in innate immunity (Hoggard et al., 2017). Antibiotics are routinely prescribed upon suspicion of bacterial rhinosinusitis. However antibiotics are only effective in a small subset of patients and inflammation triggered by fungal/viral infection or exposure to irritants is symptomatically indistinguishable (Kaplan, 2014). Poor antimicrobial stewardship continues to burden the healthcare system and contributes to the increasing prevalence of multidrug resistance in opportunistic pathogens like *P. aeruginosa* and *S. aureus* (Pendleton et al., 2013; Santajit and Indrawattana, 2016). Host-defense peptides (HDPs) are promising alternatives to antibiotics since they play essential roles in providing protection against pathogens and modulating immune responses (Hancock and Sahl, 2006; Haney and Hancock, 2013). Short synthetic analogs of naturally occurring host-defense peptides, also known as innate defense regulator (IDR) peptides (such as IDR-1018 and IDR-1002), have previously showed promise as broad spectrum anti-infectives, anti-biofilm agents immune modulators and anti-inflammatories in various animal models (Hancock and Sahl, 2006; Haney and Hancock, 2013; Wu et al., 2017; Pletzer et al., 2018).

Preclinical animal studies are essential to our understanding of complex host-pathogen interactions and the efficacy of antimicrobial and immunomodulatory therapies. Unfortunately, current murine models of acute bacterial rhinosinusitis have contributed poorly to our understanding of early sinonasal colonization, since such infections are often highly invasive and/or ethically or technically challenging (Lux et al., 2019). Here, we report a simple murine model utilizing bioluminescent and chemiluminescent imaging in live mice, allowing non-invasive monitoring of bacterial infections and the corresponding host response over time in the sinus cavity. This murine model overcomes certain limitations of those previously described since it is technically straightforward, reliable, reproducible and enables application of imaging technologies. We further adapted the model for longer-term infections by using an epidemic clinical isolate that persists in the nasal cavities and elicits prolonged inflammation. This model enabled proof of concept studies to investigate the effectiveness of several HDPs (IDR-1018, DJK-5 and IDR-1002), showing that these peptides were able to provide protection against *P. aeruginosa* or *S. aureus* mediated infections by reducing bacterial burden in the nasal cavities and/or modulating the immune responses of the mice.

## Materials and Methods

### Key Resources Table

**Table:** 

Resource	Source	Identifier or Details
**Bacterial Strains**		
*S. aureus* USA300	(CDC, 2003)	Community-acquired methicillin resistant *S. aureus*, parental strain
*S. aureus* USA300-*Lux*	(Plaut et al., 2013)	pRP1190::*luxCDABE* (*gapA-*Pro, Cm^r^)
*P. aeruginosa* LESB58	(Cheng et al., 1996)	Liverpool epidemic strain (LES)B58, isolated from a cystic fibrosis patient
*P. aeruginosa* LESB58-*Lux*	(Pletzer et al., 2018)	pUCP::lux*CDABE* (*rpoZ*-Pro, Gm^r^)
*P. aeruginosa* LESB65	(Carter et al., 2010)	LESB65, isolated from a cystic fibrosis patient
*P. aeruginosa* LESB65-*Lux*	This study	Chromosomal::*luxCDABE* (*P1*-Pro, Gm^r^)
**Recombinant DNA/plasmids**		
pUC-mini-Gm-*lux-P1*-Pro	(Damron et al., 2013)	pUC18T::mini-Tn7T::*luxCDABE*::*P1*-Pro, Gm^r^
pTNS2	(Choi et al., 2005)	pTNS::*lacI*-Pro::RSF, Kan^r^
**Chemicals and Peptides**		
IDR-1018	(Achtman et al., 2012)	VRLIVAVRIWRR-NH_2_
IDR-1002	(Nijnik et al., 2010)	VQRWLIVWRIRK-NH_2_
DJK-5	(de la Fuente-Nunez et al., 2015)	vqwrairvrvir-NH_2_
L-012 sodium salt	Millipore Sigma	Cat#SML2236
Sodium alginate	Sigma	Cat#W201502
TMB solution	eBioscience	Cat#00-4201-56
Avidin horseradish peroxidase	eBioscience	Cat#18-4100
Cytotoxicity detection kit	Roche	Cat#11644793001
**Antibodies pairs for ELISA**		
Murine IL-6	eBioscience	Cat #14-7061, 13-7062
Murine KC (CXCL1)	R&D	Cat#MAB453, BAF275
Human IL-6	eBioscience	Cat#14-7069, 13-7068
Human IL-8	Invitrogen	Cat#AHC0982, AHC0789
**Tissue Culture Reagents**		
MEM	Gibco	Cat#11090081
L-glutamine (200mM)	Gibco	Cat#25030081
Trypsin-EDTA (0.25%)	Gibco	Cat#25200056
PBS (pH7.4)	Gibco	Cat#10010023
**Experimental Models: Mouse Strains**		
CD-1 mice	Charles Rivers Laboratories, Inc	Crl : CD1(IGS)
C57Bl/6 mice	Charles Rivers Laboratories, Inc	C57Bl/6:6NCrl
**Software and Algorithms**		
GraphPad Prism 8.0	La Jolla, CA	v8.0
KC4	Bio-Tek Instruments, VT	v3.0
Living Image Lumina Series	PerkinElmer, MA	v3.1

### Bacterial Strains and Plasmids

All bacterial strains used in this study are identified in the *Key Resources Table.*

#### Bacterial Growth Conditions

Bacterial strains were streaked onto Lennox Broth (LB) agar plates from frozen stocks and grown overnight at 37*°*C. The following day, an individual colony was used to make an overnight culture in LB or 2x yeast extract-tryptone (2xYT) by incubating bacteria at 37°C with shaking (250 rpm) for no longer than 18 h. Bacterial growth was monitored by measuring optical density at 600 nm (OD_600_) with a spectrophotometer (Eppendorf, Missisauga, ON). Sub-cultures were obtained by diluting overnight cultures to OD_600_ = 0.1, and grown to an OD_600_ = 2.0 for *S. aureus* or 1.0 for *P. aeruginosa* strains. Bacterial cells were washed twice with sterile phosphate-buffered saline (PBS) and resuspended in the appropriate media to the concentrations indicated in figure legends. For plasmid selection, the following antibiotics were used: 50 μg/ml chloramphenicol (Cm) for *S. aureus* USA300-Lux, and 500 μg/ml gentamicin (Gm) for *P. aeruginosa* LESB58-Lux.

#### Generation of a *P. aeruginosa* Chromosomally-Tagged Bioluminescence Strain

Plasmid pUC-mini-Gm-lux-P1-Pro (Damron et al., 2013) was co-electroporated with helper plasmid pTNS2 (Choi et al., 2005) into *P. aeruginosa* LESB65 cells as described earlier (Pletzer et al., 2016). Briefly, bacteria were scraped from an agar plate grown overnight and resuspended in 300 mM sucrose prior to washing twice. After the last washing step, the pellet was resuspended in 100 µl of 300 mM sucrose and mixed with 500 ng of each plasmid. Cells were transformed via electroporation using Electroporator 2510 (Eppendorf) 2510 at 2.5 kV, 25 μF, 200 Ω. All steps were carried out at room temperature. Cells were recovered for 3 h at 37°C in 2xYT broth with shaking at 220 rpm after electroporation. Positive clones, showing strong bioluminescence, were selected on LB agar plates containing Gm and further verified for correct chromosomal insertion via PCR of the flanking regions with transposon- and chromosome-specific primers, as described previously (Choi et al., 2005; Choi and Schweizer, 2006).

### Murine Model of Sinusitis Infection

#### Animal Care and Ethics

Animal experiments were performed in accordance with the Canadian Council on Animal Care (CCAC) guidelines and were approved by the University of British Columbia Animal Care Committee (protocol A17-0253). Mice used in this study were outbred C57Bl/6 mice (female, aged 11–13 weeks) or CD-1 mice (female, aged 7–9 weeks). All animals were purchased from Charles River Laboratories, Inc. (Wilmington, MA). C57Bl/6 mice weighed 20 ± 5 g at the time of experiment, whereas CD-1 mice weighed 25 ± 5 g. Animals were group housed in cohorts of 4-5 littermates exposed to the same bacterial pathogen. Littermates of the same sex were randomly assigned to experimental groups. Standard animal husbandry protocols were employed.

#### Intranasal Infection

Bacterial subcultures were washed twice with sterile PBS and resuspended at an OD_600_ of 1.0. For longer-term infection, LESB65 was washed twice with sterile PBS and then resuspended in sodium alginate (11 mg/ml) (Wuerth et al., 2017). 20 μl of bacteria were instilled, dropwise, into the left naris of mice under anesthesia (2.5% isoflurane) yielding inocula or 10^6^ CFU or 10^7^ CFU for *P. aeruginosa* or *S. aureus* infections, respectively. Animals were monitored and given heat support immediately following the infection. All animals were allowed to recover from anesthesia before returning them to their cage. Experiments were repeated 2-4 with 2-3 animals per group. The welfare of mice was monitored by recording clinical score, capturing changes in weight, fur, activity, hydration, breathing, and pain using a mouse grimace scale, at 3-, 24-, 48-, and 72- h post-infection for the acute infection model, while additional monitoring was provided at 120 h for the longer term infection.

#### Tracking Bioluminescent Bacteria During Infection

Disease progression was followed by acquiring bacterial bioluminescence images (60 or 90 s exposure, medium binning) under anesthesia (2.5% isoflurane) at 24- or 48-h intervals, up to 120 h post-infection, using a Lumina *in vivo* imaging system (IVIS) (PerkinElmer, Waltham, MA). The *luxCDABE* operon from *Photorhabdus luminescens* encodes both luciferase and the enzymes required for endogenous synthesis of the luciferase substrate (Damron et al., 2013). This substrate yielded a luminescent signal that was detected by assessing absorbance at 490 nm. The signal was automatically corrected for background luminescence. The luminescence image was then overlaid on the photographic image and analyzed using Living Image software 3.1 (PerkinElmer, Walthma, MA). The total number of luminescent pixels in the region of interest (ROI), normalized to its size, was expressed in arbitrary units (AU) as radiance.

#### Tracking Reactive Oxygen and Nitrogen Species Elicited to the Site of Infection

To detect the production of reactive oxygen and nitrogen species (ROS/RNS) generated during the innate immune response of the host, a chemiluminescent probe, L-012 (25 mg/kg, Millipore Sigma, Burlington, MA) that has high sensitivity to superoxide and peroxynitrite anions, was used (Kielland et al., 2009). The probe was subcutaneously injected between the ears of the mice at various time points during the course of the infection. The detection of signal was optimal within 20 ± 2 min of probe injection. Background noise is indicated as the limit of detection (LOD). Representative images were acquired using a Lumina IVIS (60 s exposure, medium binning) and analyzed using Living Image software.

#### Sample Collection

At experimental endpoint, mice were euthanized by intraperitoneal injection of sodium pentobarbital (120 mg/kg), followed by cardiac puncture for blood collection, nasal lavage, and excision of lungs and heads. For nasal lavage, an incision was made one-third of the way down the trachea and one ml of PBS was rinsed through the sinus cavity using an intravenous catheter. For lung tissues, whole lungs were collected in sterile PBS, and homogenized using a Mini Beadbeater-96 cell disrupter (BioSpec Products, Oklahoma, USA) for 5 min. Serial dilutions of nasal lavage fluid and lung homogenate were plated on LB agar for bacterial enumeration, and the remaining liquid was stored at -20°C for use in protein quantification and ELISA.

For histopathological studies of the nasal cavity, mice were decapitated and the head was degloved for optimal perfusion of fixative (10% buffered formalin) into the tissue. Decalcification, cross-sectioning and histochemical staining with hematoxylin and eosin (H&E), of samples were adapted from Lindsay et al. (2006) and performed by Wax-It Histology Services Inc. (University of British Columbia, Vancouver, CA) or ourselves. Histological evaluation was performed on samples post-infection, and representative images are shown.

#### Peptide Treatments

Peptides IDR-1018 (VRLIVAVRIWRR-NH_2_) (Achtman et al., 2012), DJK-5 (all D-amino acids vqwrairvrvir-NH_2_) (de la Fuente-Nunez et al., 2015) and IDR-1002 (VQRWLIVWRIRK-NH_2_) (Nijnik et al., 2010), were synthesized by CPC Scientific or GenScript using solid-phase 9-flurenylmethoxy carbonyl (Fmoc) chemistry and purified to >95% purity using reverse-phase high-performance liquid chromatography (HPLC). The lyophilized peptides were resuspended in endotoxin-free water.

All peptides used for treatment of intranasal infection were tested for toxicity prior to efficacy testing. Peptides were diluted in endotoxin-free water to their desired concentration with consideration of net peptide content (approximately 70% of dry weight). For shorter-term infection, peptides or water (10 μl) were instilled directly into the left naris of mice 24 h post-infection. For the longer-term infection, peptides or water (~10–13 µl) were delivered *via* Respimat inhaler^®^ (Boehringer Ingelheim, Ridgefield, CT). Progression of disease was monitored, and sample collection was performed as described above.

### Cytotoxicity of Human Bronchial Epithelial Cells Caused by *P. aeruginosa*

The SV40-transformed, immortalized human bronchial epithelial (HBE) cell line, 16HBE14o- (Cozens et al., 1994) were cultured in minimum essential medium with Earle*’*s salts (MEM, Gibco, Massachusetts, USA) containing 10% heat-inactivated fetal bovine serum (FBS) (Gibco) and 1% L-glutamine (Gibco), at 37*°*C in a 5% CO_2_ humidified incubator. To passage cells, medium was removed and the cells were washed with PBS. Adherent cells were detached with 0.25% Trypsin-EDTA (Gibco, Massachusetts, USA) at 37*°*C in a 5% CO_2_ in a humidified incubator for approximately 4–6 min. An equal volume of MEM containing 10% FBS and 1% L-glutamine was added to neutralize the reaction. Cells were centrifuged at 1,000 rpm for 5 min, and the supernatant was removed. Fresh media were used to resuspend the cells before seeding into new flasks or plates.

HBE cells were seeded into 96-well plates (~4 x 10^5^ cells/well) and incubated at 37*°*C with 5% CO_2_. Media were refreshed the next day, and cells were allowed to grow to confluency in a monolayer. On the day of infection, media were removed and replaced with MEM supplemented with 1% FBS and 1% L-glutamine, and cells were allowed to rest for at least 1 h. For each replicate, the number of HBE cells per well was determined on the day of the experiment, and the multiplicity of infection (MOI) was calculated.

Sub-cultures of *P. aeruginosa* LESB58 and LESB65 were grown to an OD_600_ = 1.0 in LB. Bacterial cells were washed twice with sterile PBS and resuspended in MEM containing 1% FBS and 1% L-glutamine. HBE cells were infected with *P. aeruginosa* LESB58 or LESB65 at a MOI of 1, 5, 10 or 100 for 18-, 24-, or 48-h. Cell free tissue culture (TC) supernatants were collected at experimental endpoints by centrifugation (4*°*C, 5 min at 1000 rpm to remove detached HBE cell and debris, supernatant was transferred to new tubes, follow by 5 min at 8000 rpm to remove bacterial cells). Cellular cytotoxicity was determined by monitoring the release of the enzyme lactate dehydrogenase (LDH) in the TC supernatant, using a colorimetric detection assay from Roche Diagnostic (Laval, QC, Canada), according to the manufacturer*’*s instructions. The remainding TC supernatants were aliquoted and stored at -20°C for protein quantification.

### Enzyme-Linked Immunosorbent Assay

Nasal lavage fluid, lung homogenates and TC supernatants were aliquoted and stored at -20°C until needed for ELISAs. Levels of cytokines and chemokines were measured using eBioscience (San Diego, California, USA) antibodies for murine TNF-α and IL-1β and human IL-6. MIP-2 and KC (CXCL1) antibodies were from R&D Systems, Inc. (Minneapolis, MN, USA). Human IL-8 was purchased from Invitrogen (Carlsbad, California, USA). Serial dilutions of the recombinant cytokines or chemokines were used to establish a standard curve for evaluation of the protein concentration in TC supernatants. ELISAs were performed following the manufacturer’s protocols with optimization of antibody and sample dilutions, washes, and incubation times. They were developed using 3,3’,5,5’-tetramethylbenzidine (TMB) solution (eBioscience) and the reaction stopped with concentrated (2 N) sulfuric acid. The plates were read on a Power Wave X340 plate-reader (Bio-Tek Instruments) and fitted to a 4-parameter standard curve using KC4 software (v3.0, Bio-Tek Instruments).

### Quantification and Statistical Analysis

Luminescent signals were quantified using Living Image software (v3.1, PerkinElmer, MA). Statistics were performed using GraphPad Prism v8.0 (La Jolla, CA). *P *values were calculated using Kruskal Wallis nonparametric test followed by Dunn*’*s post-hoc analysis, Student*’*s unpaired two-tailed t-test with Bonferroni-Dunn*’*s correction or Mann-Whitney U-test as indicated in the figure captions. Statistical significance was established as *P* < 0.05. Other statistical details of experiments can also be found in the figure captions.

## Results

### Acute Intranasal Infection

#### Using Bioluminescent and Chemiluminescent Signals to Monitor Disease Progression

**Figure 1:**
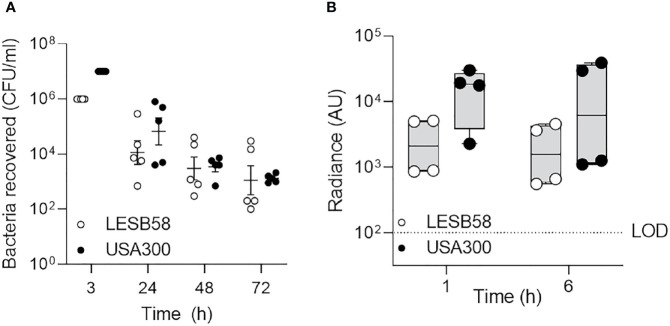
Clinically important species of bacteria established acute intranasal infection and elicited reactive oxygen species production. *P. aeruginosa* LESB58-Lux or *S. aureus* USA300-Lux were inoculated dropwise in the left naris of C57Bl/6 mice (10^6^ or 10^7^ CFU, respectively). **(A)** Bacteria were recovered up to 72-h post-infection by nasal lavage. **(B)** Localization of oxidative species to the site of infection was tracked using the chemiluminescent L-012 sodium salt probe (25 mg/kg). Radiance was quantified 1- and 6-h post-infection across species. Infection with *S. aureus* USA300 resulted in a significantly stronger oxidative response than *P. aeruginosa* LESB58 1 h post-infection. The limit of detection (LOD) is shown as a dotted line at 10^2^ counts. *n* = 4 per time point. Data are shown as mean ± SEM.

Development of murine models of rhinosinusitis has been hampered by the tendency of bacteria to disseminate or be aspirated into the lungs of the host to the extent where they cannot be reliably enumerated in the sinus cavity (Jin et al., 2011). To monitor this phenomenon in the acute rhinosinusitis model, we used bioluminescently tagged strains to follow the ability of clinical isolates *S. aureus* USA300 and *P. aeruginosa* LESB58 to initiate and maintain intranasal infection. Clinical isolates (Cheng et al., 1996; CDC, 2003) characterized by reduced virulence were selected to avert host-mediated clearance and were instilled dropwise with sufficient time between drops to allow absorption and prevent aspiration. Luminescence signals were used to monitor bacterial burden in the nasal cavity in real time (**Figure S1** ****). The bacterial burden over the course of the experiment was further quantified by obtaining colony forming unit (CFU) counts from the nasal lavage fluid (**Figure 1A**). Local inflammatory responses were assessed 6-h post-infection by monitoring the production of ROS/RNS, expressed as radiance (**Figure 1B**).

Luminescent signals were detected in the nasal cavities, but not in the lungs, of mice administered ~10^7^ CFU *S. aureus* USA300-Lux, for up to 24-h post-infection (**Figure S1A**). The amount of *S. aureus* USA300-Lux recovered by nasal lavage 24-h post-infection was ~10^5^ CFU (**Figure 1A**). Although luminescence could not be detected beyond this time point, the number of bacteria in the nasal cavity was stable for up to 3 days post-infection (8.4 ± 1.2 x 10^3^ CFU). It appears likely that the bacterial counts recovered, especially at 24-h post-infection, underestimated the numbers of bacteria present, possibly due to biofilm formation/aggregation and/or tight adherence of bacteria to tissues. To determine whether aspiration or dissemination could account for loss of luminescence, bacteria recovered from lung homogenates were examined (**Figure S2A**). Similar to the pattern observed for the nasal cavity, bacterial counts from the lungs were greatest at 24-h post-infection, with 6.2 ± 3.2 x 10^4^ CFU *S. aureus* USA300-Lux recovered. Consistent weight loss in mice over time corroborated infection persistence for at least 3 days (**Figure S2B**).

Since acute bacterial rhinosinusitis is associated with neutrophil and macrophage recruitment/activation to help eradicate invading pathogens, partially through an oxidative response (Kara, 2004), the chemiluminescent probe L-012 (8-amino-5-chloro-7-phenylpyrido[3,4-d]pyridazine-1,4(2H,3H)dione) was used to measure the production of ROS/RNS in live mice infected with non-luminescent bacteria (**Figure 1B**) (Kielland et al., 2009). Strong radiance was detected 1-h post *S. aureus* USA300-Lux infection (~1.2 x 10^4^ radiance), but the signal was more variable after 6 h (**Figure 1B**, **Figure S3A**). No signal was detected at 12 h (data not shown). The rapid decline of ROS/RNS indicated that activation of neutrophils and macrophages recruited to the site of infection was transient.

Compared to *S. aureus* USA300-Lux, mice administered with ~10^6^ CFU *P. aeruginosa* LESB58-Lux expressed stronger luminescence 24-h post-infection (**Figure S1B**) and 1.2 ± 2.1 10^4^ CFU were recovered from nasal lavage at this time (**Figure 1A**). Similar to *S. aureus*, *P. aeruginosa* LESB58-Lux luminescence could not be detected beyond this time point (**Figure S1B**), even though 7.2 ± 2.7 x 10^3^ CFU *P. aeruginosa* were recovered from nasal lavage 3 days post-infection (**Figure 1A**). From lung homogenate, bacterial counts were greatest at 24-h post *P. aeruginosa* infection (4.3 ± 2.4 x 10^3^ CFU), and the number of bacteria declined thereafter. Mice infected with *P. aeruginosa* also consistently lost weight, but overall animal welfare was stable (**Figure S2B**). When compared to *S. aureus*, *P. aeruginosa* elicited a weaker oxidative response (~2.6 x 10^3^ radiance), but ROS/RNS were more consistent over-time (**Figure 1B**). This suggested that a *P. aeruginosa* LESB strain might be amenable to establishing a longer-term infection model.

Bacterial load in the lungs depended on the density of the inoculum used to establish an infection (**Figure S4**). Mice inoculated with *P. aeruginosa* LESB58-Lux at higher densities of 10^7^ CFU and 10^8^ CFU exhibited increased luminescence in the lungs after 24 h than mice inoculated with 10^6^ CFU (**Figures S4A, B**). Additionally, mice that were treated with more dense infections survived less (**Figure S4C**). Within 24 h of a higher dose infection, 25%–50% of mice reached their clinical endpoint, whereas in the lower bacterial density cohort, all mice survived until the experimental endpoint at 72-h post-infection. Furthermore, the tendency for bacteria to be aspirated into the lungs of mice depended on host genotype (data not shown). CD-1 mice treated with *P. aeruginosa* LESB58-Lux exhibited stronger luminescence in the lungs at 3- and 24-h post-infection compared to C57Bl/6 mice treated with the same dose of bacteria. Moreover, no signal was detected in the nasal cavities of CD-1 mice 24-h post-infection. This effect was also exhibited for *S. aureus* USA300-Lux infection in CD-1 mice.

#### Bacterial Induction of Inflammation Was Partly Mediated by Neutrophils

**Figure 2:**
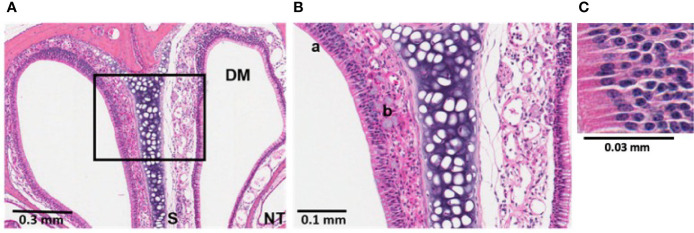
Bacterial induction of inflammation in sinonasal mucosa was partially mediated by neutrophils. Histological sections treated with hematoxylin and eosin (H&E) stain revealed sinonasal mucosa with neutrophilic inflammation that was most pronounced at 24 h post-infection. *P. aeruginosa* LESB58 was inoculated dropwise in the left naris of C57Bl/6 mice (~10^6^ CFU) providing a within-subject control in the right naris. **(A)** Focal neutrophil infiltration (30-40x more neutrophils per high power field) was observed at a 4 mm deep cross-section of the nasal cavity. Sinus secretions (mucus) with admixed cells were most abundant at 24-h post-infection. **(B, C)** Select areas of the field were expanded for visualization of a) neutrophilic inflammation and b) mucus secreting cells. Abbreviations used: DM, dorsal meatus; NT, nasoturbinate; S, septum.

Since we observed a rapid induction of reactive oxidative species in the nasal cavities of infected mice, histological investigations were conducted following *S. aureus* USA300 (**Figure S5**) or *P. aeruginosa* LESB58 infection (**Figure 2**). Examinations revealed that bacteria-mediated neutrophil infiltration was most pronounced at 24 h in tissues sectioned in 2 mm increments from the ventral nares, and was sustained for up to 72 h (**Figure 2**, **Figure S5**). As indicated in the Figure, more a) neutrophils and b) mucus secreting cells were detected in the mucosa lining the dorsal meatus (DM) of the left naris, when compared to that of the right naris that was not exposed to infection, as early as 24-h post-infection (**Figure 2**). Indeed, 30–40x more neutrophils per high powered field were enumerated in the DM of the left naris of mice exposed to either *S. aureus* or *P. aeruginosa*. Similar reactivity was observed in other clinically-relevant areas of the sinus cavity, including the ethmoid and maxillary sinuses (ES and MS, respectively) and the nasopharyngeal meatus (NPM) (**Figure S5**). Disease progression was indicated by accumulation of mucosal secretions and epithelial damage resulting from persistent reactive mucosa in these regions at 48-h post-infection. Interestingly, the left olfactory bulb (OB) of mice intranasally exposed to infection was also inflamed. Sham infection with only PBS did not induce an inflammatory response (**Figure S6**).

#### Host-Defense Peptides Reduced Bacterial Load and Stabilized Weight Loss Post-Infection

**Figure 3:**
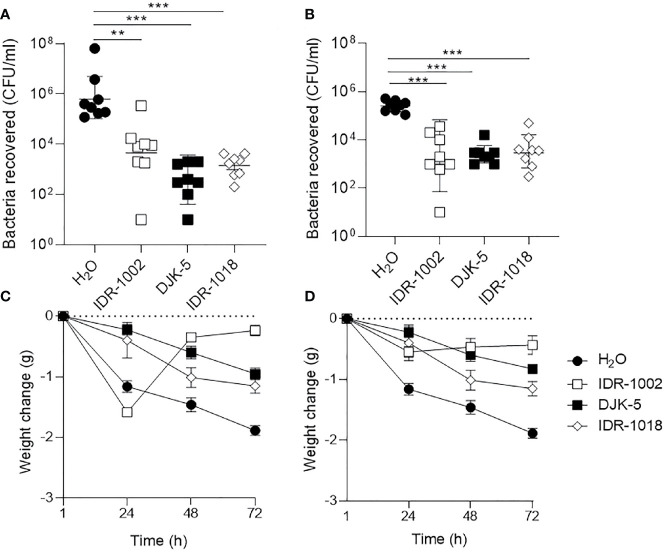
Host-defense peptides reduced bacterial load in nasal cavity and stabilize weight loss following infection. **(A, C)** *S. aureus* USA300-Lux or **(B, D)** *P. aeruginosa* LESB58-Lux were inoculated dropwise in the left naris of C57Bl/6 mice (10^7^ or 10^6^ CFU, respectively). Mice were intranasally treated with endotoxin-free water (vehicle) or peptide (7.5 mg/kg or 2.5 mg/kg for DJK-5) 24-h post-infection. Forty-eight hours later, mice were euthanized and bacteria in the nasal cavity were collected by lavage. **(A, B)** All peptides reduced bacterial burden of the sinuses approximately 100-fold across species. **(C, D)** Peptide treatment stabilized weight loss or promoted weight gain following infection across species, whereas mice treated with endotoxin-free water continued to lose weight over time. *n* = 8. Data are presented as geometric mean ± SD. **P < 0.01, ***P < 0.001 according to Mann-Whitney U-test.

The increased prevalence of multi-drug resistant strains of pathogens implicated in acute bacterial rhinosinusitis, and the low effectiveness of conventional antibiotics, warrant efficacy assessment of alternative therapies in this context (Rezai et al., 2016). Here, we showed that synthetic HDPs IDR-1018 (7.5 mg/kg), DJK-5 (2.5 mg/kg), and IDR-1002 (7.5 mg/kg) each reduced bacterial burden of the nasal cavity and improved the overall welfare of mice when administered dropwise into the naris 24 h after infection (**Figure 3**). Peptides IDR-1018 and DJK-5 exhibited more potent antibacterial activity than IDR-1002 in the context of *S. aureus* USA300 infection (**Figure 3A**). Peptides IDR-1018 and DJK-5 significantly decreased *S. aureus* bacterial burden by ~4,275-fold and ~10,500-fold, respectively, while IDR-1002 showed a lesser and non-significant decrease compared to the vehicle control. Compared to the control group, IDR-1018 and DJK-5 also prevented weight loss in the initial 24 h in mice infected with *S. aureus*, and were able to reduce overall weight loss (**Figure 3C**). Interestingly, treatment with IDR-1002 did not prevent the initial weight lost at 24-h post-infection, but mice treated with the peptide regained most of the weight by 48-h post-infection. All peptide treatments exhibited similar antimicrobial activity in the context of *P. aeruginosa* infection, by significantly reducing bacterial burden 25- to 100-fold (**Figure 3B**). In addition, all peptide treatments were able to reduce weight loss in the initial 24-h post-infection, without additional intervention (**Figure 3D**).

Intranasal administration of a small volume of peptides impacted lung topology. Peptides (7.5 mg/kg for IDR peptides and 2.5 mg/kg for DJK-5) did not cause lesioning or accumulation of precipitate in the lungs of infected mice (**Figure S7**), and were less punctate or discolored than lungs of infected mice treated with water only. Taken together, these results indicated that peptides were able to protect against bacterial mediated damage in the lungs of mice through antimicrobial and potentially immunomodulatory activity.

### Long-Term Intranasal Infection

To adapt the acute infection model for the study of long-term infections, an appropriate bacterial strain that mediated persistent infection and inflammation without inducing significant toxicity to the host was first identified. It has been reported that the *P. aeruginosa* LESB are particularly successful as pathogens in being able to establish and out-compete other strains during chronic respiratory infections of CF patients (Fothergill et al., 2012). Here, we examined the use of LESB58 and LESB65 in a longer-term sinusitis infection model.

#### *P. aeruginosa* Clinical Isolate for Long-Term Infection Was Identified

**Figure 4:**
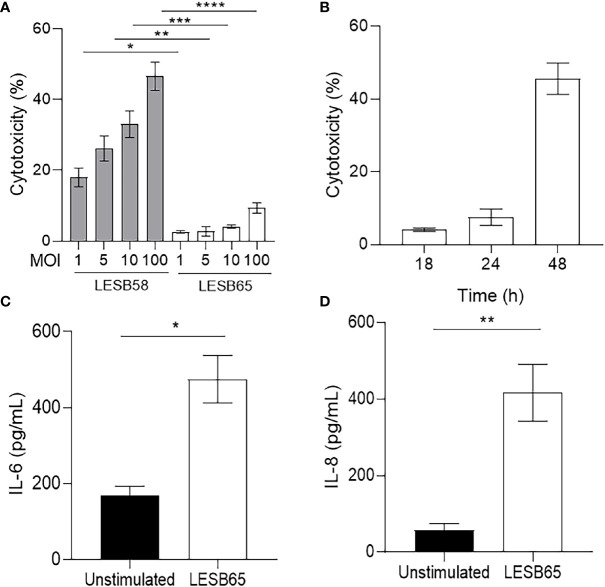
Host response to *P. aeruginosa* LESB strains *in vitro*. **(A)** Human bronchial epithelial cells (HBE) were infected with *P. aeruginosa *LESB58 or LESB65 at a multiplicity of infection (MOI) of 1, 5, 10, or 100 for 18 h. Amount of LDH in host-cell supernatants was quantified for determination of cytotoxicity. **(B)** At MOI = 10, *P. aeruginosa *LESB65 took 48 h to elicit cytotoxicity similar to that caused in 18 h by LESB58. Furthermore,* P. aeruginosa *LESB65 (MOI = 10) mediated significant production of **(C)** pro-inflammatory cytokine (IL-6) and **(D)** chemokine (IL-8) by HBE cells 18-h post-infection. Data are shown as mean ± SEM. *P < 0.05, **P < 0.01, ***P < 0.001, ****P < 0.0001 according to Student’s t-test.

The cytotoxicity against HBE cells of LESB58 and LESB65 was examined to identify an appropriate *P. aeruginosa* isolate for longer-term infection. Bacterial mediated cytokine responses of HBE cells were also measured (**Figure 4**). To compare the degree of virulence, LESB58 and LESB65 were used to infect HBE cells for 18 h at a range of concentrations. LESB58 mediated a significant amount of HBE cell death in a dose-dependent manner, in contrast to strain LESB65 that exhibited minimal cytotoxicity (**Figure 4A**). LESB65 took 48 h to mediate approximately 45%–50% host cell death (**Figure 4B**), equivalent to the cytotoxicity cause by strain LESB58 in 18 h (**Figure 4A**). The growth rate of the two strains was compared to ensure that differences in virulence were not confounded. There were no differences between growth of LESB58 and LESB65 in various media including LB, DYT, and MEM with 1% FBS and 1% glutamine media (data not shown). Since LESB65 caused no significant host cell death 18-h post-infection, we investigated whether it instead influenced epithelial cell cytokine responses. ELISAs on cell supernatants revealed that LESB65 infection induced secretion of the pro-inflammatory cytokine IL-6 (**Figure 4C**) and chemokine IL-8 (**Figure 4D**) by HBE cells.

#### Using Radiance to Monitor Disease Progression

**Figure 5:**
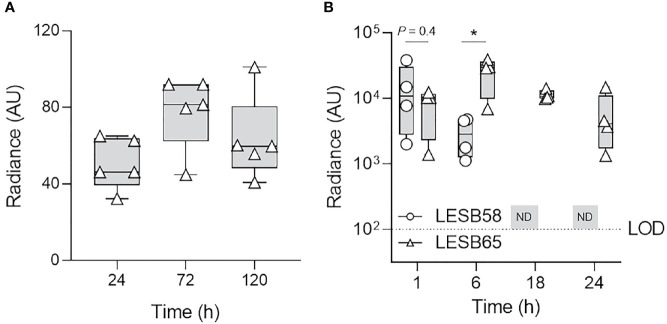
*P. aeruginosa* LESB65 persisted in the nasal cavity for up to 5 days and elicited a prolonged inflammatory response. *P. aeruginosa* LESB65-Lux encapsulated in sodium alginate (11 mg/ml) was inoculated dropwise in the left naris of C57Bl/6 mice (~10^6^ CFU). **(A)** Bacteria were detected by luminescence imaging for up to 120 h. **(B)** Localization of oxidative species to the site of LESB58 or LESB65 infection was tracked using the chemiluminescent L-012 sodium salt probe (25 mg/kg). No difference in the mean intensity of the signal was detected until 6 h post-infection, at which time the signal was stronger in mice treated with LESB65. The signal was not detected (ND) in mice treated with LESB58 beyond this time point. *n* = 4-5. Data are shown as geometric mean ± SD. The limit of detection (LOD) is shown as a dotted line at 10^2^ counts. *P < 0.05 according to Student’s t-test.

To mimic the alginate-containing biofilm environment during chronic infection, *P. aeruginosa* LESB65-Lux was encapsulated in sodium alginate (11 mg/ml) and inoculated in the left naris of mice (~10^6^ CFU) and tracked for up to 5 days (**Figure 5**, **Figure S8**). Since bacteria were encapsulated in alginate, nasal lavage did not adequately rinse bacteria from the nasal cavity of mice (data not shown). Instead, radiance was used to estimate the relative bacterial burden in the sinus cavity over time (**Figure 5A**). Radiance fluctuated slightly, but insignificantly, over the course of infection and was maintained for up to 120-h post-infection. The median radiance measured in mice 72-h post-infection (78.0 AU) was slightly higher than 24-h post-infection (50.5 AU) and 120-h post-infection (63.5 AU).

To determine whether *P. aeruginosa* strain LESB65 elicited prolonged inflammation in the nasal cavity compared to LESB58, the production of ROS/RNS was again measured using the chemiluminescent L-012 probe in live mice (**Figure 5B**). ROS/RNS in the nasal cavity of mice infected with strain LESB65 was similar to that of strain LESB58 1-h post-infection (**Figure 5B**), which indicated a rapid, robust host oxidative response. However, unlike *P. aeruginosa* strain LESB58, the signal obtained following strain LESB65 infection continued to increase for up to 6-h post-infection and could still be detected 24-h post-infection (**Figure 5B**). Radiance detected in the nasal cavity of mice infected with LESB58 was stable for up to 6 h but could not be detected at later time points of 18 and 24 h (**Figure 5B**). At 6 h post-infection, the intensity of the signal detected following LESB65 infection was significantly higher (~10-fold) than that following LESB58 infection. Overall, these results indicate that intranasal infection of LESB65 in alginate sustained a prolonged host inflammatory response.

#### Histology Confirmed Prolonged *P. aeruginosa* LESB65 Mediated Inflammation

**Figure 6:**
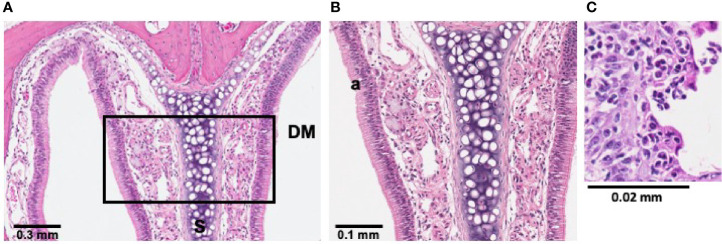
LESB65-mediated inflammation was maintained in sinonasal mucosa for 5 days. *P. aeruginosa* LESB65 was inoculated dropwise into the left naris of C57Bl/6 mice (~10^6^ CFU). Histological sections treated with hematoxylin and eosin (H&E) stain revealed that sinonasal mucosa with neutrophilic inflammation was maintained 5 days post-infection. **(A)** Immune cell infiltration was observed at a 4 mm deep cross-section of the nasal cavity. **(B, C)** Select areas of the field were expanded to enable visualization increased neutrophil infiltration (a).

To verify persistence of LESB65-mediated inflammation, histological sections of mouse nasal cavities were collected 5 days post-infection (**Figure 6**). H&E stain revealed LESB65-mediated inflammation in the nasal cavity 5 days post-infection, with increased neutrophil infiltration, pus and mucus production in the left naris of mice (**Figures 6B, C**).

#### Host-Defense Peptides Enhanced Recovery of *P. aeruginosa* LESB65 Infected Mice

**Figure 7:**
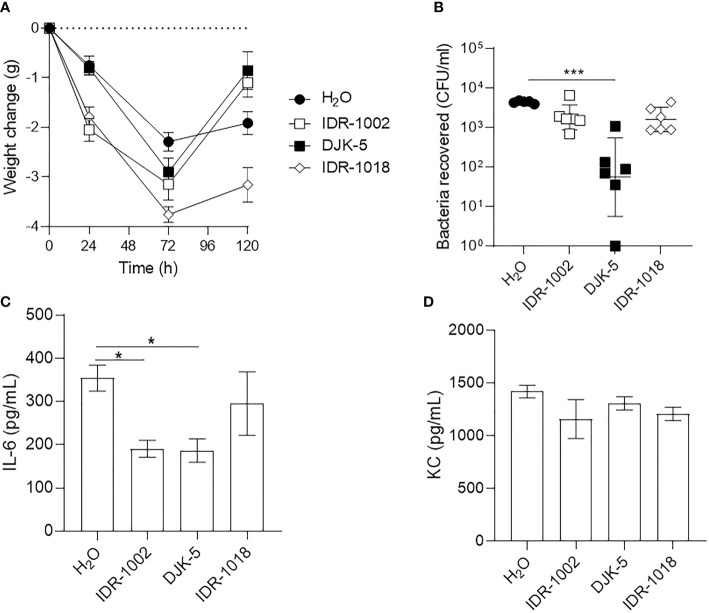
Host-defense peptides exhibited immunomodulatory and/or antimicrobial activities during LESB65 infected mice. *P. aeruginosa* LESB65-Lux encapsulated in alginate were inoculated dropwise in the left naris of C57Bl/6 mice (~10^6^ CFU). Mice were treated with endotoxin-free H_2_O (vehicle) or peptide (1.25 mg/kg) using a Respimat device 24-h post-infection. **(A)** Weight was recorded at 0-, 24-, 72-, and 120- h post-infection, and mean weight change from day 0 are reported. Lung homogenates were used to **(B)** enumerate bacterial burden and determine the production of **(C)** pro-inflammatory cytokine (IL-6) and **(D)** chemokine (KC/CXCL1). **(A, B)** Data are shown as geometric mean ± SD or **(C, D)** mean ± SEM. *n* ≥ 6. *P < 0.05, ***P < 0.001 according to Kruskal-Wallis test followed by Dunn’s correction.

To further validate the longer-term infection, murine weight change, bacterial titre in the lungs and bacterial mediated immune responses were monitored in the presence and absence of host-defense peptides administered by Respimat device (1.25 mg/kg) (**Figure 7**). Mice treated with peptides lost weight more rapidly than mice treated with the vehicle during the first 72 h. Mice treated with IDR-1002 or DJK-5 recovered weight by 120 h, better than mice treated with IDR-1018 (**Figure 7A**). Luminescence emitted from LESB65-*Lux* could be monitored to qualitatively estimate peptide activity, or peptide-mediated reduction of bacterial load, but was not used for quantification since luminescence fluctuated over time in the absence of peptide treatment (**Figure S9**). Further, since nasal lavage did not adequately rinse bacteria from the nasal cavity of mice, only bacterial load from the lungs was enumerated. Similar to shorter-term infection, treatment with IDR-1018 and DJK-5 significantly reduced bacterial load in the lung, but IDR-1002 demonstrated no significant reduction (**Figure 7B**). We further assessed effects of peptides on LESB65-mediated inflammation in the lung. Both peptides IDR-1002 and DJK-5, but not IDR-1018, signifcantly suppressed pro-inflammatory cytokine IL-6, without effecting KC production (**Figures 7C, D**). Taken together, these results suggested that peptides exerted distinct activity during *P. aeruginosa* LESB65 infections.

## Discussion

The multifactorial etiology of rhinosinusitis was inadequately understood until recent elucidation of the role of inflammation and likely microbial biofilms in more chronic cases, which are characterized by persistent sinonasal immune responses (Lee and Lane, 2011). The mucosal epithelium lining the sinus cavity plays an important role in rhinosinusitis, since it is the first point of contact between the host and pathogens in which various immune signaling cascades are initiated (Hoggard et al., 2017; Lux et al., 2019). Here, we describe a preclinical murine model of acute bacterial sinusitis to study pathological mechanisms underlying rhinosinusitis and disease progression. Although the sinus anatomy of mice is not identical to that of humans, the epithelial architecture is the same (Al-Sayed et al., 2017). More specifically, the anterior naris is lined by a stratified, non-ciliated epithelium whereas the rest of the nasal cavity is lined by a ciliated columnar epithelium in both mice and humans (Weidenmaier et al., 2012). Thus, immune cell infiltration and inflammation associated with rhinosinusitis, as well as efficacy of peptides at combatting these properties, can be examined in this model.

In the rhinosinusitis model established here, the C57Bl/6 murine nasal cavity was colonized with clinical isolates of two common pathogens found in rhinosinusitis and in CF patients, *S. aureus* (USA300) and *P. aeruginosa* (LESB58 or LESB65) (**Figure 1**, **Figure S1**, **Figure 5**, **Figure S8**). Both strains of bacteria were able to be maintained in nasal cavity for at least 72 h, without compromising the welfare of the animals. Moreover, strong inflammatory responses were elicited, and immune cell infiltration was sustained in the nasal epithelium of mice for the duration of the infection (**Figures 2**, **6**, **Figure S5**). In similar models, inflammation could not be achieved by acute infection in the absence of nasal obstruction, immune system compromisation, or antibiotic therapy (Kruszewska et al., 2004; Jin et al., 2011). The model described here is, therefore, more representative of the natural acquisition of bacteria from the environment via the nasal (inhaled) route and the early stages of disease pathogenesis. Moreover, this model is more straightforward to execute and less injurious to the host than those previously described. A combination of factors might have contributed to the differences observed in our model including the bacterial strains used, density of inocula and mouse genotype. For example, *S. aureus* strain Newman is a different clinical isolate that is used extensively in animal models due to its robust virulence phenotypes (Sakr et al., 2018). However, use of this strain is associated with rapid clearance from the nasal cavity, which was partially attributed to competition with resident nasal flora and clearance by immune cells stimulated by microbial enterotoxins (Xu et al., 2015). Interestingly, *S. aureus* strains that were originally isolated from humans, including strains Newman and Reynolds, colonized the nares of mice no better than those originally isolated from mice such as strain DAK (Kiser et al., 1999). Pre-exposure to agents that interfere with immune processes and community interactions enable stable colonization of the murine nasopharynx, but limit the ability to subsequently test treatment efficacy. Thus, we turned to strains that have been successfully used in a chronic localized abscess model (Pletzer et al., 2018). USA300 is a slightly less virulent community-acquired epidemic strain of methicillin resistant *S. aureus* (MRSA) that is able to persist on mucosal surfaces without aberrant immune activation at low density, which might be one reason for the suitability of this strain in establishing nasal colonization (Brown et al., 2014). Compared to the common *P. aeruginosa* laboratory strains, PAO1 and PA14, epidemic CF clinical isolate LESB58 demonstrates reduced swimming motility, which might lead to reduced dissemination from the nasal cavity to the lungs (Sousa and Pereira, 2014). Importantly, CF lung isolates exhibit little-to-no expression of flagellar genes, suggesting reduced motility as a feature contributing to pathogenesis and lack of invasiveness in CF (Jeukens et al., 2014). Furthermore, the LESB58 strain produces more biofilm than PAO1 and PA14 (Kukavica-Ibrulj et al., 2008). These features might be particularly important in the context of respiratory disorders, especially in CF patients in which mucociliary defense mechanisms are compromised. Further work is needed to characterize the bacterial factors that influence the carriage of pathogenic strains and identify targets of immunity that influence colonization patterns.

Towards this end, our model was amended to study a longer-term infection using a less virulent chronic infection CF isolate, *P. aeruginosa* LESB65 (**Figures 4**–**7**) (Salunkhe et al., 2005). Although LESB58 and LESB65 produced similar levels of virulence factors such as pyocyanin *in vitro*, LESB65 caused greater damage to the lungs of mice in a model of acute pneumonia (Carter et al., 2010; Jeukens et al., 2014). To mimic the matrix-protected biofilm environment, and to recapitulate mucus build-up in the airways of sinusitis and CF patients, LESB65 was encapsulated in sodium alginate. Others have reported that infections with LESB65 resuspended in PBS solution can last from 4 days (in BALB/c mice, with 60% of the mice surviving) up to 28 days (in BALB/cOlaHsd mice; survival rate not reported) (Carter et al., 2010; Fothergill et al., 2014). The model described herein allowed infections to persist for at least 5 days. Notably, all mice survived until the experimental endpoint. Although special preparation of inocula added a layer of complexity to the protocol, encapsulation of bacteria did not mitigate host immune responses (**Figures 5** **–** **7**). However, alginate interfered with recovery of bacteria by nasal lavage. To overcome this, bacterial load was estimated by measuring radiance arising from luminescent bacteria *in situ* (**Figure 5A**, **Figure S8**). Interestingly, bacterial luminescence could be maintained for up to 120 h post-infection (**Figure 5A**, **Figure S8**). *P. aeruginosa* LESB65-mediated inflammation in the nasal cavity, as indicated by ROS/RNS generation, pathology and cytokine production, was also maintained up to 120 h (**Figure 5B**).

Compared to other published models, the model described herein allows non-invasive tracking of rhinosinusitis progression (**Figures 1**, **5**). Time-course assessments of bacterial load and host response to acute bacterial rhinosinusitis have previously relied on progressive sacrifice of different animals and are limited by the instability of reactive metabolites in lavage fluid (Kara, 2004; Frieri et al., 2015; Lux et al., 2019). The use of our technique not only reduced the variability associated with physiological differences between subjects, which in turn allowed more controlled analysis of disease progression, but also reduced cost and were arguably more ethical (Rai and Kaushik, 2018). Loss of luminescence in the nasal cavity of mice infected with bacteria that were not encapsulated in alginate could be attributed to downregulated activity at the Lux-tagged promoter below the limit of detection, which was dictated by light scattering and absorption of surrounding tissue (Choi et al., 2005), or to oxygen limitation that impacts on luminescence. ROS/RNS was associated with inflammatory disease progression with kinetics typical of what is usually observed when neutrophils and macrophages rapidly infiltrate sites of infection to execute innate immune functions (Rodriguez et al., 2007), peaking in the first hours following infection (**Figures 1**, **2**, **5**, **6**). Bacterial retention in the nasal cavity despite neutrophilic influx and an oxidative burst further suggested that the model could be applied to study longer-term infection circumstances. Interestingly, the oxidative burst elicited by LESB65 infection was more pronounced and sustained at later time points than that elicited by LESB58 infection (**Figure 5**). Further work is needed to determine the bacterial and host factors that contribute to the sustained host response.

HDPs and their synthetic analogs are promising antimicrobial and immunomodulatory agents that inhibit biofilm formation *in vitro*, as well as reduce abscess size and modulate inflammation *in vivo* (Overhage et al., 2008; Wu et al., 2017; Pletzer et al., 2018; Alford et al., 2020). We showed that the synthetic HDPs DJK-5, IDR-1002, and IDR-1018 effectively reduced bacterial load in the nasal cavities of mice exposed to USA300, LESB58, and/or LESB65 infections and prevented weight loss associated with disease progression without toxic side effects in the lungs (**Figures 3**, **7**, **Figure S7**). Interestingly, the peptides prevented extensive punctation and discoloration of the lungs associated with lesions caused by bacterial colonization (**Figure S7**). Since bacteria can rapidly form biofilms to cope with harsh conditions within the host, treatment administered 24-h post-infection *via* intranasal installation likely did not wash bacteria into the lungs (Overhage et al., 2008; Wuerth et al., 2017). Administration of peptides IDR-1002 and DJK-5 reduced bacterial-mediated inflammatory responses in the lung, while maintaining induced chemokine expression and promoting weight recovery. More specifically, peptide therapy reduced the levels of the pro-inflammatory cytokine IL-6, overexpression of which has been associated with development of nasal polyps in chronic rhinosinusitis (Min and Lee, 2000), without reducing the chemokine KC, which is important for neutrophil recruitment to the site of infection (Min and Lee, 2000). These data suggest that the peptides have the ability to reduce inflammation, while maintaining the ability to recruit immune cells to the lung, and enhance recovery of mice (**Figure 7**).

Validation of animal models used for efficacy testing is essential for bridging the gap between pre-clinical and clinical development of emerging therapies for infectious diseases. The murine model of bacterial rhinosinusitis described herein can be used to assess the antibacterial, antibiofilm and/or immunomodulatory activity of promising therapeutic candidates and can result in better translation to human treatment. Since chronic rhinosinusitis is the most common clinical form of this disease (Meltzer and Hamilos, 2011; Hoggard et al., 2017) and administration of therapies 24 h after infection is not clinically feasible, future studies should involve longer term infection with intervention at more relevant time points. The model described at present can easily be adapted for the study of longer-term infections in both the sinuses and the lungs and contribute to our understanding of pathogenic mechanisms associated with initial rhinosinusitis infection or recurrent pulmonary exacerbations in CF.

## Data Availability Statement

The original contributions presented in the study are included in the article/**Supplementary Material**. Further inquiries can be directed to the corresponding author.

## Ethics Statement

Animal experiments were performed in accordance with the Canadian Council on Animal Care (CCAC) guidelines and were approved by the University of British Columbia Animal Care Committee (protocol A17-0253).

## Author Contributions

MA and K-YC were responsible for conceptualization, investigation, validation, visualization and interpretation of data, formal analysis, drafting, and editing of the manuscript. MT provided significant mentorship and guidance in conception of the project. HM performed all of the histology in a blinded fashion. PK assisted in optimizing and comparing growth conditions between LESB58 and LESB65 using HBE cells. DP created the *P. aeruginosa* LESB58 and 65-Lux strains. RH was responsible for conceptualization, funding acquisition, provision of resources and supervision, project administration and editing of the manuscript. All authors contributed to the article and approved the submitted version.

## Funding

We gratefully acknowledge funding to RH from the Canadian Institutes for Health Research grant FDN-154287 and Michael Smith Foundation for Health Research grant 17774. MA holds a UBC Killam Doctoral Scholarship and CIHR Vanier Graduate Scholarship. K-YC is supported by the Michael Smith Foundation for Health Research and Lotte & John Hecht Memorial Foundation Research Trainee Award. MT is supported by the Michael Smith Foundation for Health Research. REWH holds a Canada Research Chair and a UBC Killam Professorship.

## Conflict of Interest

The peptides in this study were invented by RH and co-inventors, assigned to his employer, the University of British Columbia, filed for patent protection, and licensed to ABT Innovations Ltd. in which RH has an ownership position.

The remaining authors declare that the research was conducted in the absence of any commercial or financial relationships that could be construed as a potential conflict of interest.
