# Supplementary material for: Murine Model of Sinusitis Infection for Screening Antimicrobial and Immunomodulatory Therapies
Source: Front Cell Infect Microbiol. 2021 Mar 12;11:621081. doi: 10.3389/fcimb.2021.621081 (PMC7994591; doi:10.3389/fcimb.2021.621081)
Supplement: Supplementary file 1 [file DataSheet_1.docx]

Supplementary Material

# Supplementary Data

## Supplementary Figures

**Supplementary Figure 1.** **Clinically important species of bacteria established intranasal infection. (**A) *S. aureus* USA300-Lux or (B) *P. aeruginosa* LESB58-Lux were inoculated dropwise in the left naris of C57Bl/6 mice (10^7^ or 10^6^ CFU, respectively). Mice were imaged using an *in vivo* imaging system (IVIS) for a maximum of 3 days, but no signal was detected after 24 hours. *n* = 4 per time point. One representative image is shown.

**Supplementary Figure 2. Aspiration or dissemination of bacteria from the nasal cavity did not account for loss of luminescence but could have contributed to weight loss following infection.** *S. aureus* USA300-Lux or *P. aeruginosa* LESB58-Lux were inoculated dropwise in the left naris of C57Bl/6 mice (10^7^ or 10^6^ CFU, respectively). Weight was recorded each day following infection. (A) Lung tissue was harvested in 1 ml PBS, homogenized, diluted and plated on LB for enumeration. Bacterial recovery from the lungs was greatest 24 hours post-infection. Since radiance in the nasal cavity was still detected at this time point, and bacterial load in the lungs subsequently decreased, it is unlikely that aspiration or dissemination accounted for loss of luminescence in the days following. (B) Mice infected with bacteria experienced weight loss over the course of infection but overall animal welfare was stable. Data are shown as geometric mean ± SD.

**
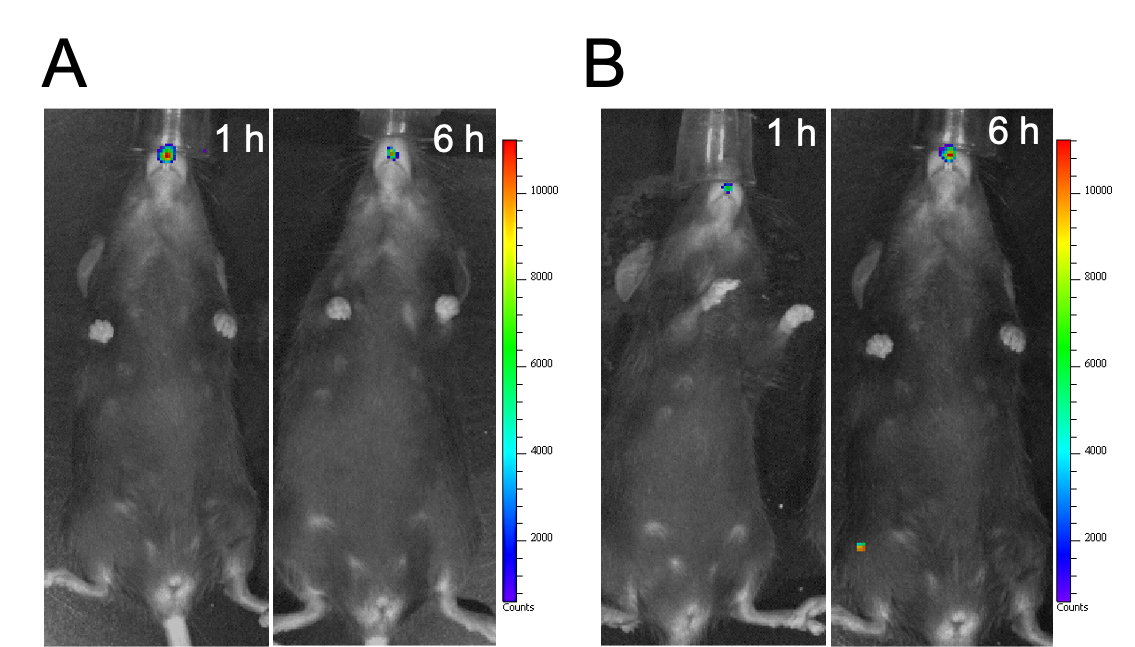
**

**Supplementary Figure 3.** **Clinically important species of bacteria elicited reactive oxygen species production in the nasal cavity.** (A) *S. aureus* USA300 or (B) *P. aeruginosa* LESB58 were inoculated dropwise in the left naris of C57Bl/6 mice (10^7^ or 10^6^ CFU, respectively). Localization of oxidative species to the site of infection was tracked using the chemiluminescent L-012 sodium salt probe (25 mg/kg). One representative image is shown.

**
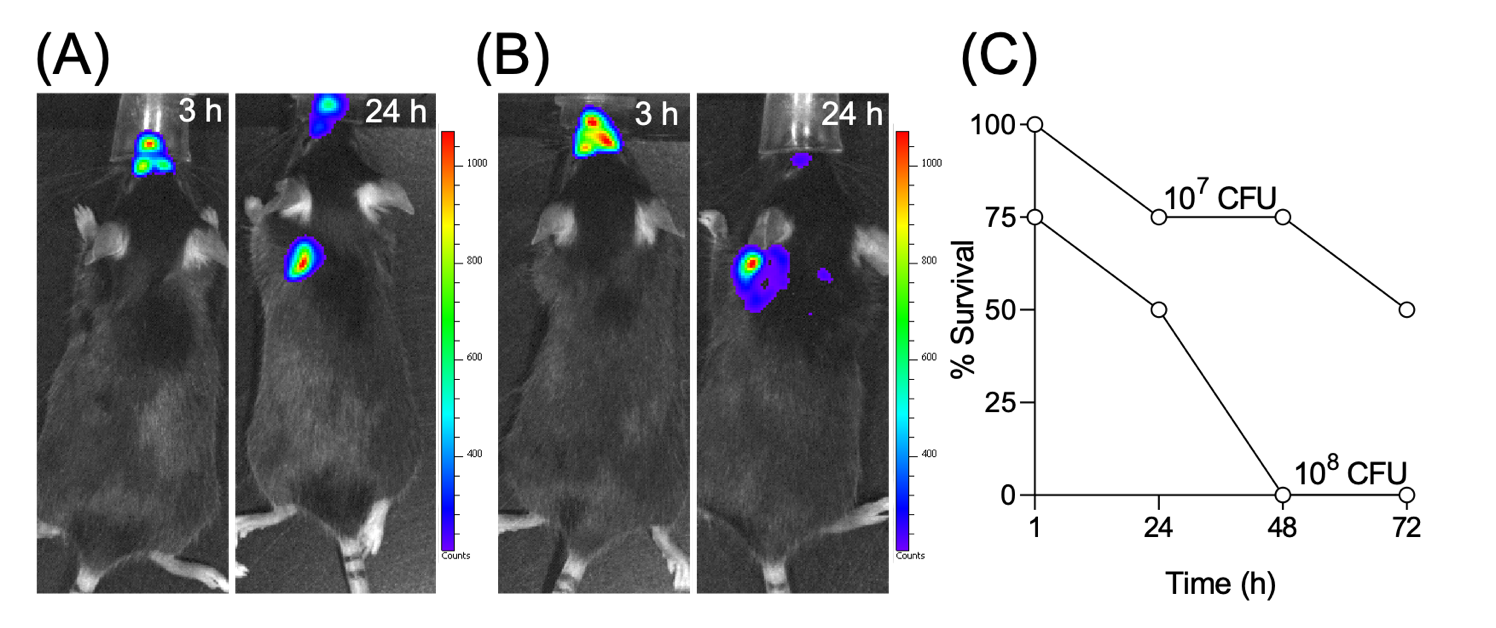
**

**Supplementary Figure 4. Aspiration or dissemination of bacteria from the nasal cavity depended on density of infection and contributed to mortality.** (A) Lux-tagged *P. aeruginosa* LESB58 was inoculated dropwise in the left naris of C57Bl/6 mice at densities of (A) 10^7^ or (B) 10^8^ CFU. Mice were imaged using an in vivo imaging system (IVIS) for a maximum of 72 hours. Extent of bacterial aspiration into the lungs was associated with density of infection. (C) Survival (%) of mice (to the humane endpoint) inoculated with bacteria at a lower dose was greater than that for mice inoculated with a higher dose of bacteria. 50% of mice in the lower density cohort, but no mice in the higher density cohort, survived to the experimental endpoint. *n* = 4. One representative image is shown.

**Supplementary Figure 5. Bacterial induction of inflammation in the murine nasal cavity was partly mediated by neutrophils.** Histological sections treated with hematoxylin and eosin stain revealed reactive mucosa that was most pronounced at 24 hours post-infection, but sustained up to 72 hours. *S. aureus* USA300 was inoculated dropwise in the left naris of C57Bl/6 mice (~10^7^ CFU) providing a within-subject control in the right naris. Focal neutrophil infiltration (30-40x more neutrophils per high power field) was observed at (A) 2 mm and (B) 6 mm deep cross-sections of the nasal cavity. Sinus secretions (mucus) with admixed cells were most abundant at 24 hours post-infection. Arrowheads indicate mucus producing cells and regions of inflammation. Abbreviations used: DM = dorsal meatus, ES = ethmoid sinus, ET = ethmoturbinate, LM = lateral meatus, MS = maxillary sinus, NT = nasoturbinate, NPM = nasopharyngeal meatus, NALT = nasal associated lymphoid tissue, OB = olfactory bulb, S = septum.


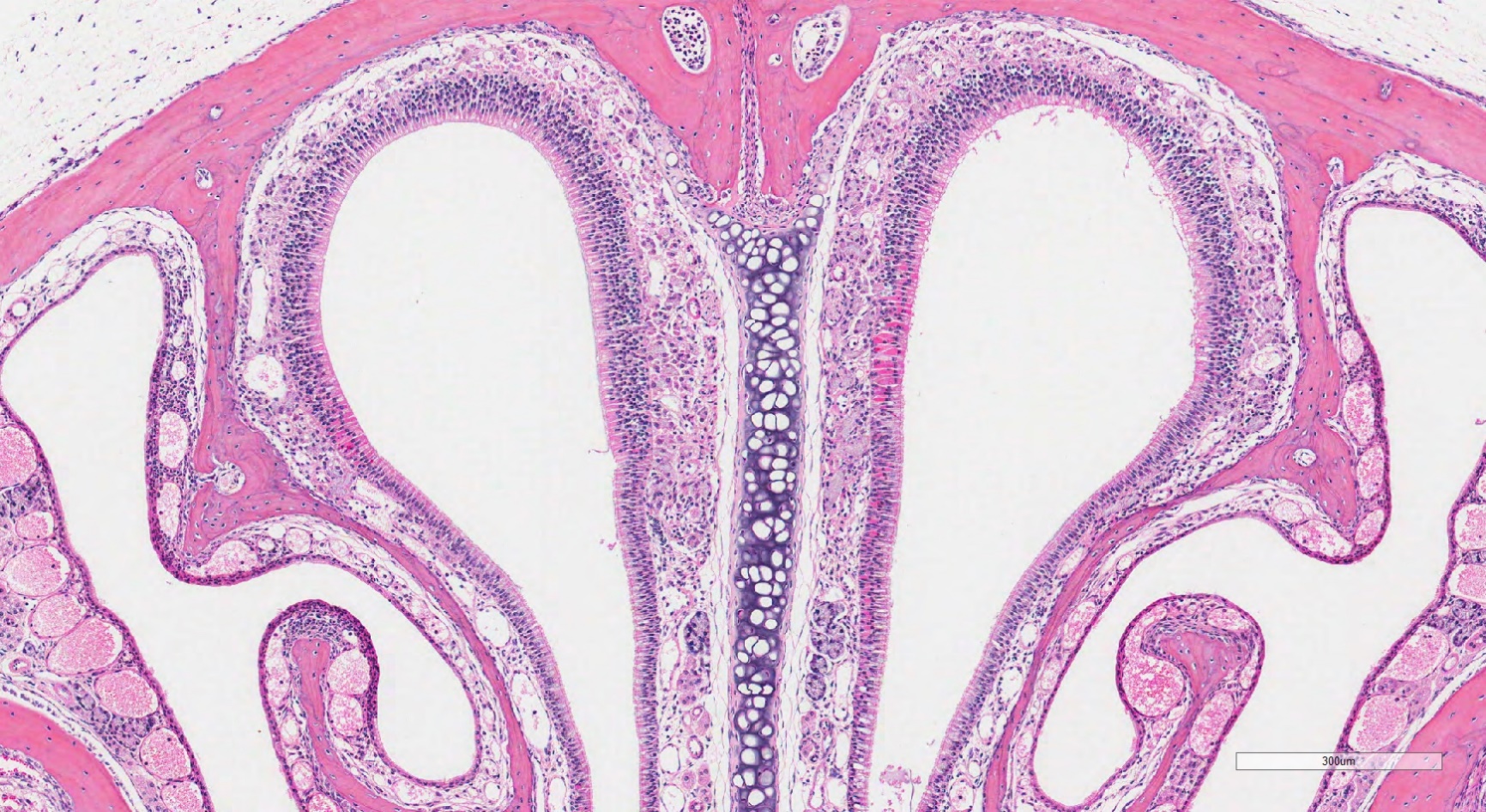


**Supplementary Figure 6. Sham infection with PBS did not induce an inflammatory response in the murine nasal cavity.** Histological sections treated with hematoxylin and eosin stain revealed no differences between PBS treated and untreated nares. PBS was instilled dropwise into the left naris of mice, providing a within-subject control in the right naris.

**
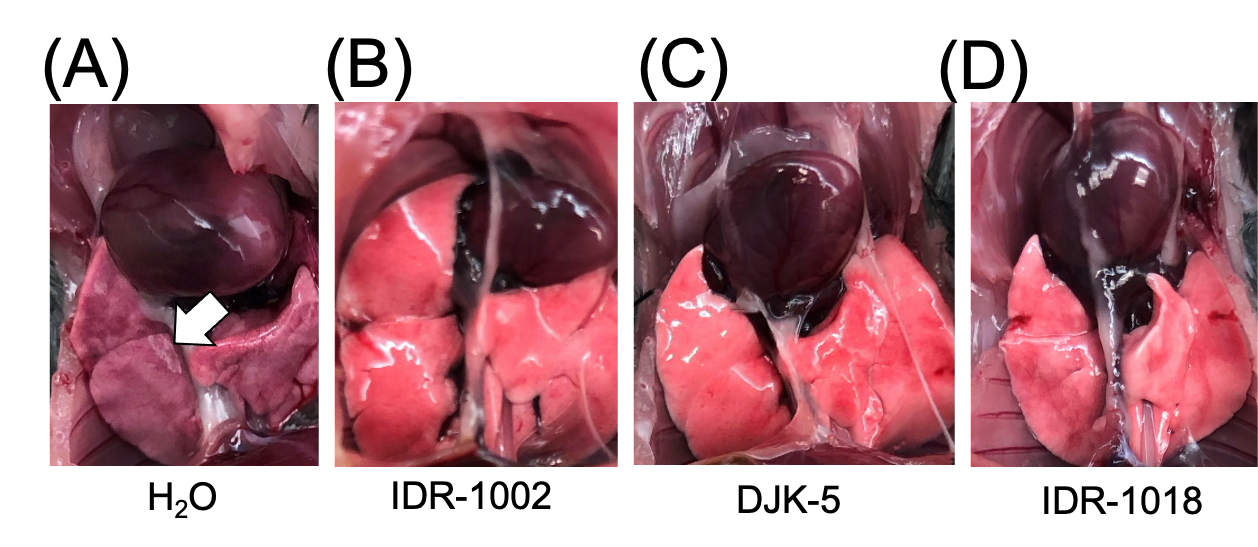
**

**Supplementary Figure 7. Host defense peptides prevent lung lesions caused by bacterial dissemination or aspiration from sinus infection in situ.** *P. aeruginosa* LESB58 was inoculated dropwise in the left naris of C57Bl/6 mice (~10^6^ CFU). 24 hours post-infection, mice were intranasally treated with endotoxin-free H_2_O (vehicle) or peptide (2.5–7.5 mg/kg). 48 hours later, mice were euthanized and lung tissue was evaluated for lesions. Bacterial aggregation was observed in lungs treated with H_2_O only, as denoted by the arrowhead. One representative image is shown.

**Supplementary Figure 8. A less virulent clinical isolate of *P. aeruginosa* persisted in the murine nasal cavity for up to five days.** *P. aeruginosa* LESB65-Lux encapsulated in sodium alginate (11 mg/ml) was inoculated dropwise in the left naris of C57Bl/6 mice (~10^6^ CFU). Mice were imaged using an *in vivo* imaging system (IVIS). One representative image is shown.

**
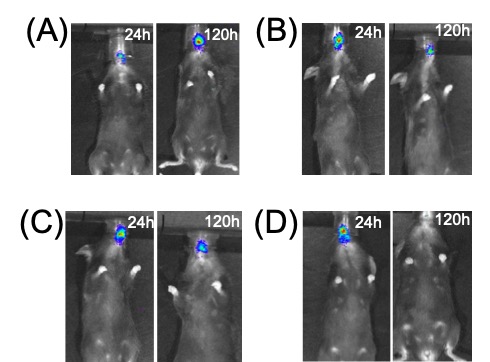
**

**Supplementary Figure 9. Peptide treatment modulated luminescence emitted by *P. aeruginosa* LESB65 in the murine nasal cavity.** *P. aeruginosa* LESB65-Lux encapsulated in sodium alginate (11 mg/ml) was inoculated dropwise in the left naris of C57Bl/6 mice (~10^6^ CFU). Mice were treated with (A) endotoxin-free H_2_O (vehicle), or peptides (B) IDR-1002, (C) DJK-5 or (D) IDR-1018 using a Respimat device 24 hours post-infection. Mice were imaged using an *in vivo* imaging system (IVIS). Representative images are shown.
